# CD39 expression on immune cells predicts methotrexate response in rheumatoid arthritis patients

**DOI:** 10.55730/1300-0144.5672

**Published:** 2023-09-09

**Authors:** Barış BORAL, İbrahim TUNCER, Filiz KİBAR, Salih ÇETİNER, Suade Özlem BADAK, Emrah SALMAN, Emrah KOÇ, Eren ERKEN, Akgün YAMAN

**Affiliations:** 1 Department of Immunology, Adana Health Practice and Research Center, University of Health Sciences, Adana, Turkiye; 2 Department of Immunology, Prof. Dr. Cemil Taşcıoğlu City Hospital, University of Health Sciences, İstanbul, Turkiye; 3 Department of Medical Microbiology, Faculty of Medicine, Çukurova University, Adana, Turkiye; 4 Department of Immunology, Faculty of Medicine, Çukurova University, Adana, Turkiye; 5 Division of Rheumatology, Department of Internal Medicine, Adana City Training and Research Hospital, Adana Turkiye; 6 Department of Immunology, Ankara City Hospital, University of Health Sciences, Ankara, Turkiye; 7 Division of Rheumatology, Department of Internal Medicine, Faculty of Medicine, Çukurova University, Adana, Turkiye

**Keywords:** Rheumatoid arthritis, methotrexate, treatment response, individualized treatment, adenosine pathway, CD39

## Abstract

**Background/aim:**

Rheumatoid arthritis (RA) is a chronic inflammatory disease affecting mostly small joints, such as hand and foot joints symmetrically with irreversible joint destruction. In this study, the relationship between CD39 expression and the treatment response of RA patients was examined to investigate its potential as a biomarker that demonstrates treatment response.

**Materials and methods:**

This study included 77 RA patients and 40 healthy controls (HC). The RA patients were divided into 2 groups based on their response to RA treatment, those with a good response to methotrexate (MTX) monotherapy and those with an inadequate response based on the American College of Rheumatology and the European League Against Rheumatism response criteria. Various immunological parameters and Disease Activity Score in 28 Joints (DAS28) were examined between the groups using the Student’s t-test.

**Results:**

The monocytic myeloid-derived suppressor cell (M-MDSC) percentage was higher in the RA patient group versus the HC group. The CD39 expression in the T lymphocytes were higher in patients that responded well to the MTX compared to those showing inadequate response. Additionally, s negative correlation was found between the DAS28 and CD39 in the T cells.

**Conclusion:**

The results showed that the improvement in treatment response to the therapy in RA patients could be because of the enhancement in the CD39/adenosine (ADO) pathway. Therefore, therapies targeting the CD39/ADO pathway in T cells may improve RA treatments.

## 1. Introduction

Rheumatoid arthritis (RA) is a chronic inflammatory disease affecting mostly small joints such as hand and foot joints symmetrically. RA causes irreversible joint destruction, which results from a rapid increase in synovial fluid and discharge of proinflammatory mediators [1,2]. To prevent irreversible joint damage, it is important to get the synovitis in RA under control with an early and effective treatment [3,4]. There is not one effective treatment for all RA patients and therefore, choosing the right disease-modifying antirheumatic drugs (DMARDs) for each patient in a timely manner has an extreme importance.

Methotrexate (MTX) is the most favored initial DMARD for patients due to its high efficacy and low toxicity, especially when compared to other conventional DMARDs (cDMARDs) [5,6]. However, some RA patients are resistant to MTX treatment. Identifying a biomarker to detect MTX treatment response as early as possible is valuable because then those patients can receive alternative effective therapies without delay [7].

**Figure 1:**
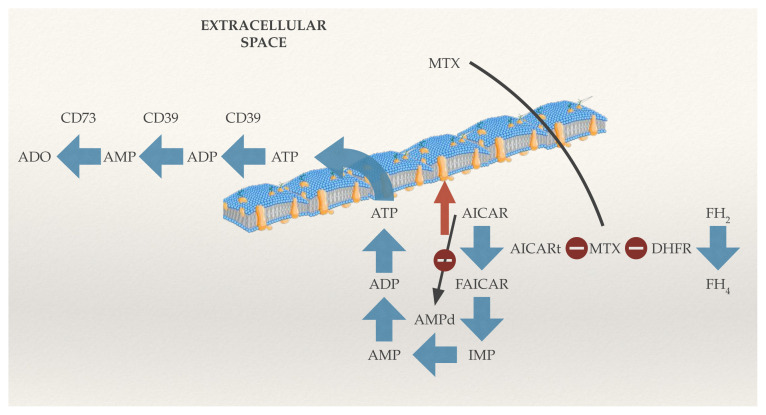
The antiinflammatory mechanism of MTX. MTX moves from the extracellular environment into the cell and inhibits the DHFR enzyme. In addition to its antifolate effect, it inhibits the enzyme AICARt, leading to AICAR accumulation. This accumulation leads to the ATP release to the extracellular compartment. The CD39 hydrolyzes both ATP and ADP into AMP. Subsequently, the CD73 degrades AMP to ADO. AICAR: 5-aminoimidazole-4-carboxamide ribonucleotide, AICARt: AICAR transformylase, FAICAR: formyl AICAR, FH_2_: dihydrofolate, FH_4_: tetrahydrofolate, ATP: adenosine triphosphate, ADP: adenosine diphosphate, AMP: adenosine monophosphate, AMPd: AMP deaminase, IMP: inosine monophosphate, MTX: methotrexate. The plasma membrane image was taken from https://www.shutterstock.com/tr/image-vector/education-chart-biology-plasma-membrane-diagram-1057812974 https://www.shutterstock.com/ and modified.

In this study, to identify a biomarker to detect MTX treatment response, the ecto-nucleoside triphosphate diphosphohydrolase (CD39) expression in immune system cells of peripheral blood was examined. CD39 can be a potential biomarker because MTX, in addition to its antifolate effect, also decreases inflammation in joints by providing high levels of extracellular adenosine (ADO) [8,9]. The occurrence of high levels of ADO happens as MTX inhibits the enzyme 5-aminoimidazole-4-carboxamide ribonucleotide (AICAR) transformylase, leading to AICAR accumulation, which in turn leads to adenosine triphosphate (ATP) release to the extracellular compartment. CD39 hydrolyzes both ATP and adenosine diphosphate (ADP) into adenosine monophosphate (AMP). Subsequently, the ecto5′-nucleotidase (CD73) converts AMP to ADO [10,11]. ADO decreases the effector functions of T lymphocytes while escalating the suppressive properties of T regulatory cells [12,13]. Furthermore, ADO can build the humoral immune response and plays a significant role in immunoglobulin class switch DNA recombination [14]. The antiinflammatory mechanism of MTX is shown in Figure 1. Therefore, CD39 can be a biomarker of MTX treatment response since it results in ADO generation.

Myeloid-derived suppressor cells (MDSCs) are myeloid precursor cells that develop from bone marrow and contain many different cell groups such as monocytic MDSC (M-MDSC), granulocytic MDSC, and immature MDSC. They suppress other immune system cells to prevent excessive immune response [15]. To achieve that, MDSCs may produce highly immunosuppressive mediator such as reactive oxygen species, indolamine-2-3-dioxygenase, nitric oxide oxidase, transforming growth factor-beta, and interleukin 10 (IL-10) [16,17]. MDSCs can be detected in peripheral blood, lymphoid tissue, cancerous tissue, and inflammation sites, and the peripheral blood was examined in this study. Previously, studies have examined the role of MDSCs during RA progression. Their findings showing the antiinflammatory behavior of MDSC suggests that it can be important for autoimmune diseases [18].

In this study, the main goal was to determine the effect of CD39 expressed by MDSCs, and B and T lymphocytes on MTX resistance and investigate potential biomarkers that predict MTX resistance.

## 2. Materials and methods

### 2.1. Study groups

This study included 77 RA patients and 40 healthy controls (HC). The youngest and oldest individuals from the study populations were 33 and 71 years old, respectively. They all provided written informed consent to be included in the study. The RA patients were screened between April 2017 and November 2020. Their blood samples were collected before receiving treatment. The RA patients were diagnosed based on the American College of Rheumatology and the European League Against Rheumatism (EULAR) criteria [19].

The RA patients were divided into 2 groups based on their response to MTX monotherapy. All of the patients received MTX monotherapy for at least 3 months. RA treatment response was defined based on the EULAR criteria [20]. The Disease Activity Score in 28 Joints (DAS28) scores were measured at the beginning and after a 3-month treatment period. The measurements were taken between 3 to 6 months depending on the patient follow-up visits. The DAS28 scores provided the RA disease activity and were calculated using C-reactive protein [18]. A decrease in the DAS28 value of more than 1.2 from the beginning of the treatment to the 3 months after and the DAS28 value at the end of the treatment lower than 3.2 were defined as good responders (MTX-res). Those with a DAS28 value greater than 3.2 at the end of the 3-month treatment or a change in DAS28 value less than 1.2 were included in the MTX inadequate responder (MTX-IR) group [20]. RA patients were removed from the study if they had ongoing infections or if they were receiving treatment with glucocorticoid. For the patients whose pretreatment DAS28 scores were missing, if their posttreatment DAS28 scores were lower than 3.2, they were excluded from the study since enough information was not obtained to categorize them. On the other hand, if their posttreatment DAS28 scores were higher than 3.2, they were included in the study as a patient in the MTX-IR group. For the treatment of patients, MTX was administered orally with a dose of 15 mg each week.

The study protocol adhered to the requirements of the Declaration of Helsinki. The protocol was approved by the Çukurova University Ethics Committee. Informed consent was obtained from all of the individuals involved in the study.

### 2.2. Rheumatoid factor (RF) and anticyclic citrullinated peptide (ACPA) antibodies determination

ACPA antibodies in the serum samples of 77 patients with a diagnosis of RA were detected with the ARCHITECT anti-CCP assay (Abbott Laboratories, Abbott Park, IL, USA) and RF was evaluated by nephelometry (Immage 800, Beckman-Coulter, USA).

### 2.3. Flow cytometry

**Figure 2:**
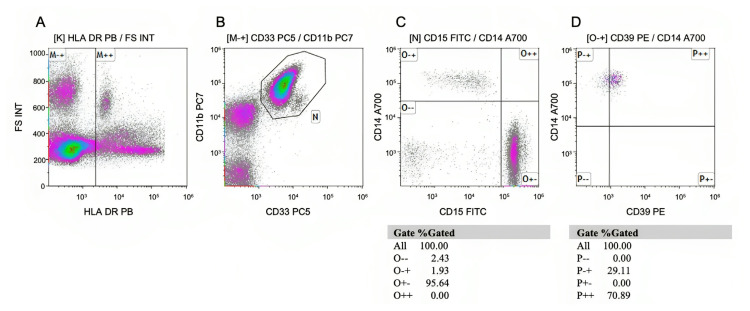
Gating strategies for the identification of CD14+ HLA-DR^low^CD11b+CD33+ MDSC subsets in the peripheral blood. Doublets were excluded. Live peripheric blood cells were gated (not shown). A) HLA-DR^low^ cells were gated. B) CD11b+/CD33+ cells were gated. C) CD14+ HLA-DR^low^CD11b+CD33+ M-MDSCs were evaluated. D) The percentage of the CD39+ M-MDSCs values are calculated among the live M-MDSCs+ cells.

**Figure 3:**
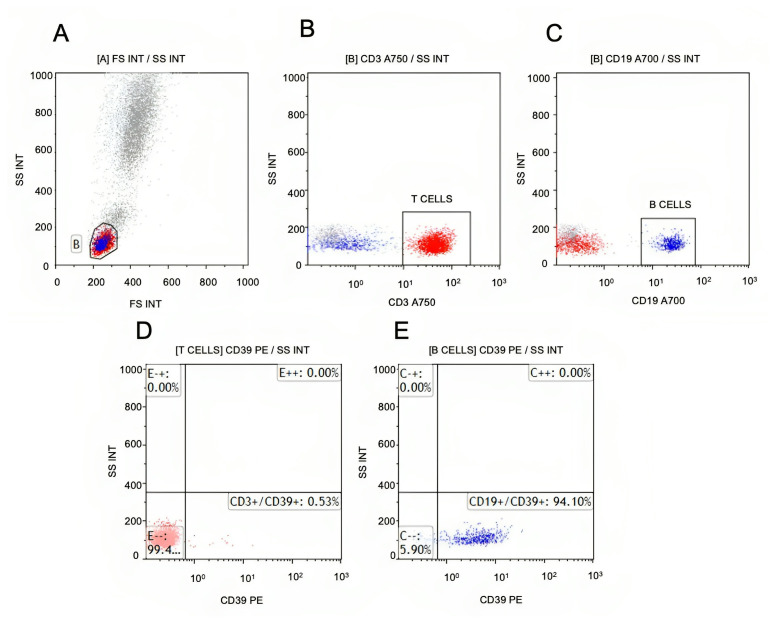
Gating strategy for CD39+ T-cells and B-cells. Doublets were excluded. Live peripheric blood cells were gated (not shown). A) Lymphocyte was gated. B) CD3+ cells were gated. C) CD19+ cells were gated. D) The percentage of CD39+CD3+ values are calculated among the live CD3+ cells. E) The percentage of the CD39+CD19+ values are calculated among the live CD19+ cells.

Peripheral blood samples were collected during the treatment naïve period and examined by flow cytometry to determine the CD33+/CD11b+/CD15+/CD14+/HLA-DR^low^ myeloid-derived suppressor cells (M-MDSCs), CD3+/CD39+ T cells, and CD19+/CD39+ B cells phenotypically. The gating strategy for the M-MDSC is provided in Figure 2. As shown in Figure 2A, first, the HLA-DRlow cells were gated and then, the CD11b+/CD33+ cells were gated (Figure 2B). The CD14+ HLA-DRlowCD11b+CD33+ M-MDSCs were evaluated (Figure 2C). Finally, as shown in Figure 2D, the percentage of the CD39+ M-MDSCs values was calculated among the live M-MDSCs+ cells. The gating strategy for the CD39+ T-cells and B-cells is provided in Figure 3, and was as follows: the lymphocyte was gated (Figure 3A) and then, the CD3+ cells were gated (Figure 3B), followed by gating of the CD19+ cells, as shown in Figure 3C. The percentage of the CD39+CD3+ values was calculated among the live CD3+ cells, which is shown in Figure 3D. The percentage of the CD39+CD19+ values was calculated among the live CD19+ cells, as shown in Figure 3E.

The M-MDSC, CD39+ T cells, and CD39+ B cells were measured by multiple-color flow cytometry with anti-CD3-APC-A700, anti-CD33-PC5, anti-CD11b-PC7, anti-CD15-FITC, anti-CD4-APC, anti-CD8-PC7, anti-CD56-APC-A700, anti-CD16-FITC, anti-CD14-APC-A700, anti-HLA-DR-BP, and anti-CD39-PE (Beckman Coulter, Brea, CA, USA) monoclonal antibodies according to the manufacturer’s instructions. The results of flow cytometry were analyzed with Kaluza Flow Cytometry Analysis Software (Beckman Coulter).

### 2.4. Statistical analysis

For the descriptive statistics, the mean + standard deviation was used to express the parametric quantitative data, the median and range were used to express the nonparametric quantitative data, and numbers and percentages were used to express the categorical data. Parametric quantitative data were compared with the Student’s t-test. p < 0.05 was considered statistically significant. A power analysis was run with a threshold of significance of 0.05 and desired power of 0.8. Linear correlation analysis was also used. All of the data were analyzed using Scientific Computing Tools (version 1.2.3, SciPy.org) for Python (version 2.7.16, Python.org).

## 3. Results

The power analysis suggested 16 patients for each group. This study included 35 patients in the MTX-res group and 42 patients in the MTX-IR group. The median ages of the groups were 52, 47, and 50 years old for HC, MTX-res, and MTX-IR groups, respectively. The female ratio for the RA patients (MTX-res and MTX-IR) was 79% and for the HC group, it was 75%. There were no significant differences between the groups in terms of their age and sex. Demographic and clinical features of the HC and RA groups are shown in Table 1.

The RA and HC groups were compared in terms of their M-MDSCs percentages. Significant differences were found between the percentages of the M-MDSCs in the RA patient groups (combined group of MTX-res + MTX-IR) and HC group (p < 0.01).

Next, whether there was a statistical difference between the MTX-res and MTX-IR groups in terms of the CD3+CD39+, CD19+CD39+, CD33+/CD11b+/CD15+/CD14+/HLA-DR^low^/CD39+ parameters was investigated. Statistically significant results were obtained in the CD3+CD39+ percentage and mean fluorescence intensity (MFI) values between the MTX-res and MTX-IR groups (p < 0.01 and p = 0.05 respectively). In the MTX-res group, the CD39 percentage and MFI values in the T lymphocytes were higher than those the MTX-IR group.

When the CD39 percentage of M-MDSCs (CD33+/CD11b+/CD15+/CD14+/HLA-DR^low^/CD39+), B lymphocyte (CD19+CD39+), and MFI parameters were investigated, no statistically significant difference was found between the groups (Table 2). The DAS28 values were analyzed and it was seen that a lower value was obtained in the MTX-res group, which had a positive response to the MTX monotherapy (2.13 ± 0.47) compared to the MTX-IR group (3.12 ± 1.19), and a statistical difference was found between the groups (p < 0.01).

The statistical analysis of the immunological markers between MTX-res and MTX-IR groups is shown in Table 2.

**Figure 4:**
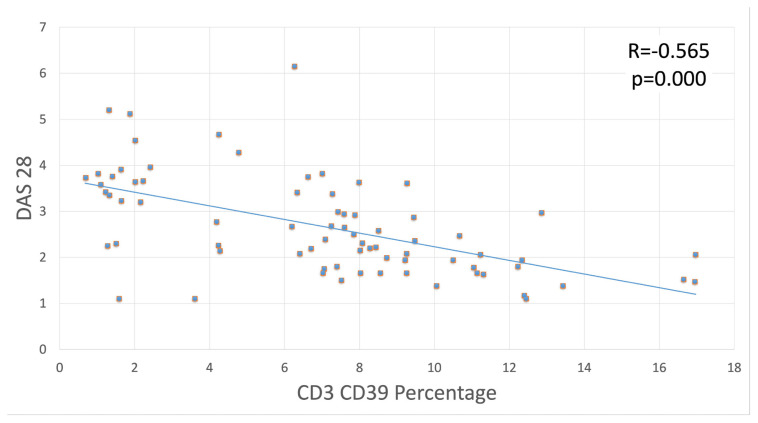
Linear correlation plot of the CD3-CD39 percentage and DAS28.

The CD39 expressions of immune regulatory cells (M-MDSCs, B, and T lymphocytes) in the peripheral blood samples of the 77 RA patients were compared with their DAS28 values (Table 3). A negative correlation was found between the DAS28 and CD3/CD39 percentage (Figure 4).

## 4. Discussion

This study demonstrated that the CD39 expression in T lymphocytes is lower in patients with an inadequate response to MTX treatment compared to those who respond well. The increase of CD39 in the T cells negatively correlates with the DAS28 and hence, CD39 in the T cells is associated with better disease activity. Studies have shown that CD39+ Treg cells have a higher suppressive capacity and suppress proinflammatory cytokines such as interferon (INF)-gamma and IL-17 than CD39-Treg cells [21–23]. Peres et al. [24] showed that unsuccessful MTX treatment in RA patients was highly correlated with a low MFI of CD39 on Treg cells. The reduction of CD39 on Treg cells impairs the production of ADO and reduces the suppressive activity of these Treg cells. Therefore, Peres et al. [24] suggested that the low MFI of CD39 on Treg cells can be a biomarker to predict MTX nonresponsiveness in RA patients. In another study, it was shown that a greater percentage of CD39+ Treg cells and CD4+ CD25+ CD39+ T cells in the peripheral blood were associated with the response to MTX in RA [23]. This is because of the strong suppressive effect of CD39+ Treg cells or the CD4+ CD25+ CD39+ T cells convert proinflammatory ATP to ADO. Therefore, both an increase in ADO production and a decrease in ATP in the extracellular environment may contribute to the immunosuppressive environment. In the study of Gupta et al., it was demonstrated that CD39 expression did not change with MTX treatment, and CD39 expression was determined genetically [23]. Another study that showed that genetic effects are important in CD39 expression found that single nucleotide polymorphism in the ENTPD1 (CD39) gene is associated with inadequate response to MTX [25]. This suggests that CD39 expression may be an important marker in predicting treatment response.

In this study, the CD39 expression in B cells was also investigated. Most of the studies examining the relationship between CD39 expression and MTX resistance have focused on T lymphocytes [23,24]. However, B lymphocytes also play an important role in the pathogenesis of autoimmune diseases through IL-10 and PD-L1 molecules [26,27]. Mature B cells express CD39 and down-regulate CD73 expression. Given the existence of CD39 and little ADO due to the lack of CD73, these B cells produce AMP in the presence of extracellular ATP [28,29]. Recently, Zacca et al. conducted a study to investigate the effect of CD39 expression in B cells on the treatment of RA [30]. They found no statistically significant difference in the frequency of CD73+CD39+, CD73-CD39+ B cell subsets, the levels of CD73, and the CD39 expression on B cells between the RA patients and healthy controls [30]. Similarly, in the present study, no statistically significant difference was found in the CD39 expression in the B lymphocytes between the MTX-res and MTX-IR groups. However, the negative correlation of the CD39 expression in the B lymphocytes with the DAS28 and RF values detected by Zacca et al. was not found in the current study. Even though no significant difference was found in the CD39 expression in the B lymphocytes between the MTX-res and MTX-IR groups, a negative correlation was found between the CD39 expression in the T lymphocytes and the DAS28.

A good understanding has been established of the immunosuppressive roles of MDSCs in tumors; however, the same statement does not hold true about their roles in autoimmune diseases. Recently, studies have been published that suggest MDSCs play an immunosuppressive role [31], as well as others that suggest MDSCs cause proinflammation and advance the RA intensity in mice by increasing Th17 cell differentiation [32]. Among these studies, Zhang et. al. [32] reported that the percentage of HLA-DR^low^/CD14+ MDSCs was higher in the peripheral blood of newly diagnosed RA patients compared to the HC group. Additionally, there were correlations detected between the MDSCs and the expansion of Th17 cells, as well as between the MDSCs and disease progression. Such correlations suggest the involvement of MDSCs in RA patients in their early stages. In this study, a statistically significant increase was found in the M-MDSC frequencies in the RA group compared to the HC group. No correlation was detected between the MDSCs and the DAS-28 score, which indicates the severity of the disease.

Peres et al. [24] reported that the CD39 expression in Treg cells is closely correlated with MTX treatment response. Considering their report, the effect of M-MDSCs on MTX resistance was examined herein, since MDSCs also have suppressor properties like Treg cells and express CD39 [33]. When the MTX-res and MTX-IR groups were compared in terms of the CD39 expressions, no significant differences were found in percentile and MFI values. However, when the relationship between the MDSCs and the response to MTX treatment was investigated, a tendency to increase was found in the ratio of M-MDSCs in the MTX-IR group compared to the MTX-res group.

In the current study, parameters such as the RF, ACPA, sex, and smoking were not correlated with the treatment response to MTX. In previous studies, the males showed a better response to MTX compared to females [34,35]. Additionally, low disease activity and no previous use of DMARDs were found to be correlated with good response to MTX. On the other hand, smoking resulted in an inadequate response to MTX. Although these factors were weakly associated with MTX response, they cannot distinguish between MTX responders and inadequate responders [34,35]. Consistent with other studies, autoantibody percentages also cannot predict MTX response [36,37].

There are other studies that have also investigated the Treg cells in RA patients [38–41]. The present study examined the CD39 expression in the T lymphocytes. Examining Treg cells is expensive and requires a difficult protocol. On the other hand, the CD39 expressions in the T lymphocytes can be studied in a routine laboratory, making it a much cheaper alternative. Therefore, it is advantageous to show the potential of CD39 expressions in the T lymphocytes as the biomarker for treatment response. This was one of the strongest aspects of this study. Additionally, the CD39 expression in the MDSCs was also examined herein, which was not done in the aforementioned studies.

In summary, the CD39 expression in the T lymphocytes was lower in patients who showed inadequate response to MTX treatment, and a negative correlation was found between the DAS28 and CD39 in the T cells. Considering that the CD39 expression does not change with treatment [23,25,30], high levels of CD39 expression in the T lymphocytes may be valuable in predicting treatment response. Given that the immunomodulatory effect mediated by CD39-expressing T cells is strongly associated with treatment response in RA, therapies targeting the CD39/ADO pathway in T cells may improve RA treatments.

There are some limitations to this study. This research studied the CD39 expression in methotrexate treatment. Another important molecule for the ADO pathway is CD73, which was not studied in the current analysis. CD73 is important because CD39 hydrolyzes both ATP and ADP into AMP. Subsequently, CD73 converts AMP to ADO. ADO decreases the effector functions of T lymphocytes, while escalating the suppressive properties of T regulatory cells. We leave the analysis of CD73 to be used as a potential biomarker for future work.

## Figures and Tables

**Table 1:** Demographic and clinical features of the healthy controls (HC) and RA patient groups.

Characteristics	HC (n = 40)	MTX-res (n = 35)	MTX-IR (n = 42)
Age: median (range)	52 (33–61)	47 (40–71)	50 (43–62)
Sex: female, n (%)	30	28 (80%)	34 (81%)
ACPA positivity, n (%)	-	28 (80%)	38 (90%)
RF positivity, n (%)	-	27 (78%)	30 (72%)
Smoking, n (%)	-	6 (17%)	11 (26%)

MTX-res: MTX responder, MTX-IR: MTX inadequate responder, ACPA: anticitrullinated protein antibodies, RF: rheumatoid factor.

**Table 2:** Statistical analysis of the immunological markers between the MTX-res and MTX-IR groups.

	MTX-res (mean/SD)	MTX-IR (mean/SD)	p-value
M-MDSC (%)	1.07 ± 1.00	1.46 ± 0.87	0.08
M-MDSC/CD39 (%)	72.5 ± 22.3	70.2 ± 18.9	0.62
M-MDSC/CD39 (MFI)	1344 ± 791	1143 ± 555	0.21
CD19/CD39 (%)	90.7 ± 4.5	89.1 ± 4.6	0.14
CD19/CD39 (MFI)	2878 ± 1007	2533 ± 791	0.11
CD3/CD39 (%)	8.86 ± 3.18	5.68 ± 4.11	<0.01
CD3/CD39 (MFI)	227 ± 29	212 ± 31	0.05

The second and third columns provide the mean values and SD for the corresponding variables given in each row. p-values were obtained with the Student’s t-test. MTX-res: MTX responder, MTX-IR: MTX inadequate responder

**Table 3:** Analysis of the correlation between the DAS28 and presented parameters.

Parameters	R (correlation) value	p-value
M-MDSC	0.061	0.600
M-MDSC/CD39 (%)	0.04	0.709
M-MDSC/CD39 (MFI)	0.026	0.821
CD19/CD39 (%)	0.097	0.413
CD19/CD39 (MFI)	0.036	0.761
CD3/CD39 (%)	−0.565	<0.01
CD3/CD39 (MFI)	−0.390	<0.01

p-values were obtained with the Student’s t-test.
